# Real-world evidence of physical activity practices and policies in Greater London primary schools: A cross-sectional survey

**DOI:** 10.1371/journal.pone.0352283

**Published:** 2026-07-10

**Authors:** Amina Benkhelfa, Bina Ram, Nancy Gullett, Mark Cunningham, Mansour Taghavi Azar Sharabiani, Miranda Pallan, Esther van Sluijs, Carolyn Summerbell, Melvyn Hillsdon, Nadia Siddiqui, Sonia Saxena

**Affiliations:** 1 School of Public Health, Imperial College London, London, United Kingdom; 2 Department of Applied Health Sciences, School of Health Sciences, College of Medicine and Health, University of Birmingham, Birmingham, United Kingdom; 3 IMS Epidemiology, School of Clinical Medicine, University of Cambridge, Cambridge, United Kingdom; 4 Department of Sport and Exercise Sciences, Durham University, Durham, United Kingdom; 5 FUSE, the Centre for Translational Research in Public Health, Newcastle University, Newcastle-upon-Tyne, United Kingdom; 6 Department of Public Health and Sport Sciences, University of Exeter, Exeter, United Kingdom; 7 Department of Education, Durham University, Durham, United Kingdom; Shandong Technology and Business University, CHINA

## Abstract

**Background:**

The World Health Organization (WHO) recommends six domains for a whole-school approach to promote physical activity (PA) levels. This approach aims to help children achieve 30 of the recommended 60 minutes of daily moderate-to-vigorous physical activity (MVPA). Evidence on PA practice and policy in school settings is limited, particularly in urban areas with high deprivation, where children’s PA levels are lowest.

**Methods:**

We developed a 20-item survey based on the WHO domains and distributed it to 1826 state-funded primary schools in all 32 Greater London boroughs between 6 November 2023 and 31 December 2024. We extracted survey items relating to each domain and calculated the percentage of schools reporting each practice.

**Results:**

Our sample of 185 schools (10.1%) included at least one school from each borough and was broadly representative of school populations in Greater London, except that responding schools were more likely to have fewer pupils eligible for free school meals. Most schools (68.5%) reported implementing at least one practice aligned with each of the six WHO domains. Specifically, 98.4% reported providing curricular physical education (Domain 1), 78.9% had an active travel plan (Domain 2), 98.4% offered PA opportunities before/after school (Domain 3), 92.4% provided opportunities for PA during breaks (Domain 4), 81.7% incorporated PA into lessons (Domain 5), and 81.5% reported supporting the inclusion of pupils with additional needs in PA opportunities (Domain 6). However, implementation within domains varied; for example, only 53.5% met the nationally recommended two hours of physical education per week, with similarly low levels for before‑school PA provision and active travel initiatives.

**Conclusion:**

Most primary schools in this large, multi-ethnic urban sample reported school policies and practices aligned with WHO domains, suggesting that a whole-school approach to creating an active school environment is feasible to implement. However, we identified areas for improvement within domains, and alignment alone may be insufficient to meaningfully increase children’s PA levels. This study provides a foundation for future research linking active school environments with children’s PA and mental health, thereby informing efforts to increase MVPA and promote equitable outcomes.

## Introduction

Regular physical activity (PA) during childhood is promoted by the World Health Organization (WHO) to support lifelong physical and mental health and wellbeing [1,2], and to prevent non-communicable diseases [3]. Reducing physical inactivity by 15% by 2030 is a global target [2,4,5]. In England, less than half (47%) of children and young people (CYP; aged 5–16 years) meet the recommended 60 minutes of moderate-to-vigorous physical activity (MVPA) per day [6]. There are also significant disparities in PA across socio-economic groups, with the least physically active children more often living in lower socio-economic households [6]. Furthermore, studies have shown differences in PA intensity between children in urban and rural areas: those living in rural areas spend less time sedentary and more time in light-intensity PA compared with children in urban areas [7,8].

Whole-school PA approaches can help primary school children (aged 5-11 years) increase their engagement in physical activity, including MVPA. Schools are considered ideal settings as most children spend at least a third of their waking hours at school on weekdays. Promoting PA in schools can help embed active behaviours during childhood [9]. Given that most children attend school, whole-school approaches also have the potential to reduce health inequalities and disparities in PA levels [10–12]. Many of the opportunities to promote PA offered by primary schools are presented as a form of play, including active play, particularly during unstructured parts of the day (e.g., break times and before/after the school day) [9,13]. These opportunities contribute to children’s physical health, and active play offers pleasure and enjoyment that may help foster better social and emotional wellbeing [14], reinforcing the importance of inclusive and equitable access to play opportunities in school. Despite public health recommendations in England that 30 of the 60 minutes of daily PA should occur during the school day [15,16], 30% of CYP are reported to engage in less than 30 minutes of MVPA while at school [6].

**Fig 1:**
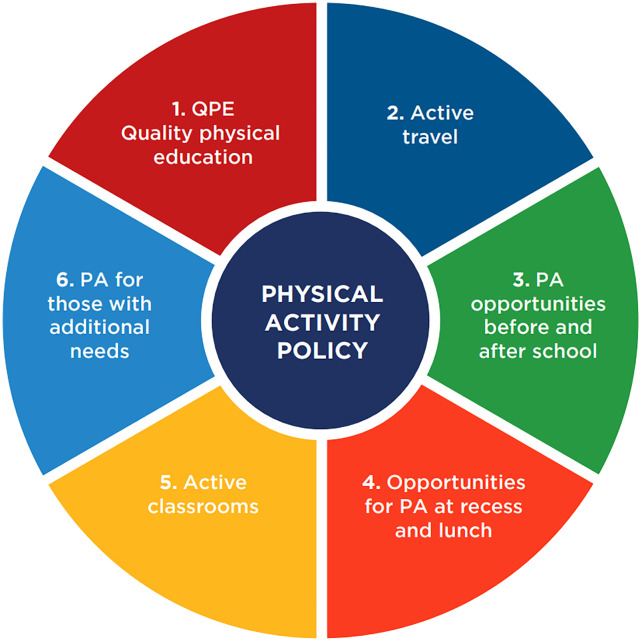
The WHO’s six domains for a whole-school approach to promote physical activity. Reproduced with permission from World Health Organization. (2021). *Promoting physical activity through schools: a toolkit*. World Health Organization. https://iris.who.int/handle/10665/350836. Published in this article under CC BY 4.0. QPE, quality physical education; PA, physical activity.

In 2021, the WHO published evidence-based guidelines for promoting PA in schools and colleges, and identified six key domains that schools should address to encourage PA among CYP [4,5]. The domains underpin a whole-school approach and are (Fig 1): (1) quality physical education (QPE), (2) active travel, (3) PA opportunities before and after school, (4) opportunities for PA at recess and lunch, (5) active classrooms, and (6) PA for those with additional needs. Further details for each domain are described in S1 Table.

Although whole-school PA promotion and its supporting frameworks have received international attention [17–19], there is limited evidence on the PA policies and practices implemented by primary schools in urban settings. Assessing current practice can reveal both strengths and barriers that are specific to diverse urban environments marked by significant health inequalities. Once identified, these findings will provide the basis for operationalising an active school environment in line with WHO guidelines, which can then be used to evaluate its effects on children’s PA levels, mental health and educational outcomes. This evidence will help inform sustainable and equitable approaches to promoting PA in schools. Hence, our study aimed to identify the extent to which primary schools in a multi-ethnic and socio-economically diverse urban conurbation of Greater London, UK, follow the WHO’s whole-school approach to PA.

## Methods

### Study design

The Health and Activity of Pupils in the Primary Years (HAPPY) study is a quasi-experimental observational study comprising two components: a quantitative school survey and a cross-sectional study. This paper presents the results of the quantitative survey using the STROBE reporting guidelines (S1 File). Details of the survey development and administration are provided below, while full methods and procedures for the broader HAPPY study, including the cross-sectional component, are available in the published study protocol [20]. Our quantitative survey targeted all state-funded primary schools in Greater London and reported on each school’s implementation of PA policies and practices in line with the WHO’s recommendations for a whole-school approach to PA, specifically assessing schools’ practices against each of the WHO’s six specified domains.

### Survey development

Our survey was developed in consultation with three primary school teachers, recruited through the NIHR Clinical Research Network, not part of the study sample, and the survey included questions adapted from previous studies [21–24]. The teachers were invited to pilot the survey through a Qualtrics link and were given one week to provide feedback. A response box under each item allowed them to comment on the ease of understanding, language, response options, overall flow, and completion time, as well as to note anything the survey did not capture but should be included. Teachers suggested minor changes, which we incorporated by clarifying one item and adding an ‘other’ option to another, and they indicated that the survey was appropriate for teacher and staff respondents.

Feedback was shared with the study team, and the final 20-item version of the survey (S2 File) was agreed upon by all authors and consulted teachers. Questions were designed with either single- or multiple-response formats, with the exception of the last question, an open-ended text box inviting optional free-text comments. The survey was made available in two formats, an online version (via Qualtrics, accessed by link or QR code) and a paper-based version. Eleven of the 20 survey questions were mapped to the WHO domains: three questions each were included under Domains 1 (QPE) and 4 (PA during scheduled breaks); two questions under Domain 5 (active classrooms); and one question each under Domains 2 (active travel), 3 (PA outside of school hours), and 6 (inclusive PA) (S2 Table).

Example questions included: “*Does your school have any of the following in place for facilitating physical activity?*” (items relating to Domains 1-5); “*When does your school (or any other organisation) provide any extracurricular physical activity or sports programmes for children at your school?*” (Domains 3 and 4); and “*Is there sufficient provision (staff/equipment) to support participation for children with specific disabilities or impairments in physical activities?*” (Domain 6). The remaining survey questions collected basic school information and broader school characteristics related to PA provision. In this paper, we report findings from the WHO domain-specific survey items only.

### Procedure

On 6 November 2023, all state-funded primary schools in Greater London (1826 at the time of this study) were sent a paper version of the survey by post, addressed to the headteacher, and included a prepaid return envelope to return the survey. For consistency, no questions were mandatory in either format. Any staff member best placed to answer the questions could complete the survey, and respondents were asked to indicate their role within the school. School contact details (headteacher name, postal address, and email address) were obtained from publicly available UK government websites [25]. To increase response rates, schools were contacted by email at three time points (January, April, and July 2024) to remind them to complete the survey. The fourth and final reminder, by post, was sent in September 2024. The survey closed on 31 December 2024.

### Statistical analysis

We compared the characteristics of responding and non-responding schools (school type, size (pupil numbers), gender, ethnic composition (based on the dominant ethnic group per school), Ofsted rating, number of pupils eligible for free school meals, number of pupils with English as an additional language, number of pupils with special educational needs and disabilities (SEND)) obtained from UK government websites [25–27]. We also compared area-level deprivation measured using borough-level quintiles of the Income Deprivation Affecting Children Index (IDACI), derived from average scores for each borough [28]. All data were categorical. Chi-squared or Fisher’s exact statistical tests were used to assess differences in characteristics between responding and non-responding schools. We report the percentage of schools that provided positive responses to each survey item included in each WHO domain. Positive responses indicated that specific practices, initiatives, or facilities were in place at the school. Responses where items were not ticked were treated as valid responses, indicating that the practice was not in place. Missing or unclassified responses were excluded from percentage calculations. All analyses were conducted using R software, version 4.4.2 [29].

### Ethics

Ethical approval was granted by Imperial College London’s Research Ethics Committee (reference: 6800895). Each school received the survey with an introductory page outlining the purpose of the study, that participation was voluntary, and assurances of confidentiality. Completion of the survey was not compulsory for the school. Formal written consent was not required to use data; return of a completed survey was considered implied consent for the data to be used for analysis. All data were anonymised for analysis (i.e., school names, locations, and teacher names were removed).

## Results

### Response rates

We received 201 responses from 1826 primary schools across all 32 Greater London boroughs, yielding an 11.0% response rate. Of these, 18 surveys were excluded from the analysis: two were from unidentifiable schools, eleven were duplicate surveys from the same school (with the most recent and complete response retained), and five lacked sufficient completion (online responses ended prematurely, typically less than 50% complete). The final sample included 185 surveys, corresponding to 10.1% of the total schools contacted. We received 115 (62.2%) survey returns by post and 70 (37.8%) through the online link. The surveys were completed by 85 (45.9%) class teachers, 74 (40.0%) headteachers or deputy headteachers, 7 (3.8%) school administrators, and 19 (10.3%) other school staff (e.g., PE/Sports Leads).

### School characteristics

Schools that completed the survey were similar in all characteristics to those that did not with the exception of the number of pupils eligible for free school meals (Table 1). Schools with more than 100 pupils eligible for free school meals were significantly underrepresented among responding schools compared to non-responding schools (*X*^*2*^ = 5.34, *p* = .021).

**Table 1:** Characteristics of Greater London schools that completed the survey compared with schools that did not.

Characteristicᵃ n (%)	Schools which completed survey (n = 185)	Schools which did not complete the survey (n = 1641)	All schools (n = 1826)	p value (schools completing survey vs. those not completing)
**Type of school¹** ^,^ **²**				
Academy/ Free	51 (27.6%)	529 (32.2%)	580 (31.8%)	
Local authority	134 (72.4%)	1112 (67.8%)	1246 (68.2%)	.196ᵇ
**Total pupil numbers¹**				
≤ 500	146 (78.9%)	1292 (78.7%)	1438 (78.8%)	
> 500	39 (21.1%)	349 (21.3%)	388 (21.2%)	.953ᵇ
**School gender¹** ^,^ **³**				
Mixed	184 (99.5%)	1632 (99.5%)	1816 (99.5%)	
Non-mixed	1 (0.5%)	8 (0.6%)	9 (0.5%)	1.000ᶜ
**Ethnic composition¹** ^,^ **⁴**				
White	113 (61.1%)	1020 (62.2%)	1133 (62.0%)	
Asian	41 (22.2%)	303 (18.5%)	344 (18.8%)	
Black	23 (12.4%)	277 (16.9%)	300 (16.4%)	
Mixed	1 (0.5%)	5 (0.3%)	6 (0.3%)	
Other	7 (3.8%)	35 (2.1%)	42 (2.3%)	.164ᶜ
**Pupils with SEND⁵**				
≤ 100	174 (94.1%)	1507 (91.8%)	1681 (92.1%)	
> 100	11 (5.9%)	134 (8.2%)	145 (7.9%)	.290ᵇ
**Pupils with English as an additional language¹**				
≤ 100	51 (27.6%)	511 (31.1%)	562 (30.8%)	
> 100	134 (72.4%)	1130 (68.9%)	1264 (69.2%)	.318ᵇ
**Pupils eligible for free school meals¹**				
≤ 100	122 (65.9%)	937 (57.1%)	1059 (58.0%)	
> 100	63 (34.1%)	704 (42.9%)	767 (42.0%)	.021ᵇ
**OFSTED rating⁶**				
Outstanding/good	177 (97.3%)	1502 (96.8%)	1679 (96.8%)	
Other	5 (2.7%)	50 (3.2%)	55 (3.2%)	.730ᵇ
**Area deprivation IDACI (quintiles)⁷**				
1 (Most deprived)	18 (9.7%)	272 (16.6%)	290 (15.9%)	
2	43 (23.2%)	393 (23.9%)	436 (23.9%)	
3	31 (16.8%)	297 (18.1%)	328 (18.0%)	
4	41 (22.2%)	331 (20.2%)	372 (20.4%)	
5 (Least deprived)	52 (28.1%)	348 (21.2%)	400 (21.9%)	.060ᵇ

ᵃPercentages are based on available data and may not total 100% due to rounding; missing/unclassified responses are excluded from percentage calculations.

ᵇChi-square test

ᶜFisher’s exact test

¹Source: https://explore-education-statistics.service.gov.uk/find-statistics/school-pupils-and-their-characteristics/2023-24

²Academy/Free Schools are Academy converter, Academy Sponsor Led and Free Schools; Local Authority Schools are Foundation, Community, Voluntary Aided, and Voluntary Controlled.

³Data not available for 1 (0.1%) school.

⁴Based on majority ethnic group per school; data not available for 1 (0.1%) school.

⁵Source: https://explore-education-statistics.service.gov.uk/find-statistics/special-educational-needs-in-england/2023-24

⁶‘Other’ includes ‘requires improvement’ and ‘special measures’; data not available for 92 (5.0%) schools; source: https://get-information-schools.service.gov.uk/

⁷Source: https://www.gov.uk/government/statistics/english-indices-of-deprivation-2019

SEND, Special Educational Needs and Disabilities; OFSTED, Office for Standards in Education, Children’s Services and Skills; IDACI, Income Deprivation Affecting Children Index

### Implementation of the WHO domains

A total of 76.0% (133/175; 10 missing responses) of schools reported having a PA policy in place, but the degree of reported implementation differed across the WHO domains (Table 2). In Domain 1 (QPE), nearly all schools (98.4%) reported delivering curricular PE, and over two-thirds (76.2%) reported access to an internal or external specialist PE teacher. However, just over half of schools (53.5%) met the curricular guidelines of providing an average of at least two hours of PE per week across year groups. In Domain 2 (active travel), 78.9% reported having an active travel plan, and 77.3% had a separate entrance for pedestrians or cyclists to encourage safe travel by foot. However, less than a quarter of schools (24.3%) promoted initiatives to support walking part of the school journey for children who travel by vehicle (‘Park and Stride’). For Domain 3 (PA opportunities outside of school hours), almost all schools (98.4%) provided PA opportunities after school, while PA opportunities before school and during weekends or holidays were less commonly reported (48.6% and 28.4% respectively). In Domain 4 (PA opportunities during scheduled breaks), 92.4% of schools reported providing PA during breaks and 82.0% during lunchtimes. Schools also reported having access to playgrounds (99.5%), portable play equipment (97.8%), access to permanent play structures (89.6%), and hardcourts (72.0%). In contrast, access to playing fields was considerably lower (48.1%). In Domain 5 (active classrooms), 81.7% of schools reported integrating PA into classroom lessons, and over half (55.1%) reported implementing active mile initiatives [30]. Lastly, in Domain 6 (inclusive PA), sufficient provision to support children with SEND was reported by 81.5% of schools. Overall, 68.5% of schools (122/158; excluding seven with missing data for one or more domain items) reported implementing all six WHO domains (at least one item under each domain).

**Table 2:** Number of schools reporting implementation of the WHO domains per corresponding survey item.

WHO domain		Criteria (survey question)	Schools meeting each item per criteria (n = 185)
1	Quality Physical Education (PE)	Do curricular PE	182 (98.4%)
		Do at least 2 hours of PE per week	99 (53.5%)
		Have access to a specialist PE teacher (internal/external)	141 (76.2%)
2	Active travel	Have an active travel plan	146 (78.9%)
		Have a ‘Park and Stride’ initiative	45 (24.3%)
		Have a separate entrance for pedestrians/cyclists from vehicles	143 (77.3%)
3	Physical activity opportunities outside school hours¹	Before school	89 (48.6%)
		After school	180 (98.4%)
		Other (weekends/holidays)	52 (28.4%)
4	Physical activity opportunities during scheduled breaks	During playtime	171 (92.4%)
		During lunch breaks¹	150 (82.0%)
		Provide access to:	
		- Playground¹	182 (99.5%)
		- Playing field¹	88 (48.1%)
		- Permanent play equipment¹	164 (89.6%)
		- Portable play equipment¹	179 (97.8%)
		- Hardcourt²	131 (72.0%)
		- Other²	23 (12.6%)
5	Active classrooms	Provide physical activity opportunities during lessons³	147 (81.7%)
		Implement physical activity initiatives (e.g., active mile interventions such as The Daily Mile, Marathon Kids)	102 (55.1%)
6	Physical activity opportunities for children with SEND	An all-inclusive approach⁴	145 (81.5%)

¹2 missing responses

²3 missing responses

³5 missing responses

⁴7 missing responses

SEND: Special Educational Needs and Disabilities

### Optional free-text comments

Just under half of responding schools provided additional free-text comments through an open-ended text box at the end of the survey, beyond what was captured by the survey questions. These included challenges in supporting physically active school environments and a lack of dedicated funding and tight budgets, which limited some schools’ ability to purchase specialised or permanent equipment, improve outdoor spaces, and subsidise extracurricular opportunities. Several schools commented that changes to the PE and Sport Premium funding criteria (a UK government fund introduced in 2013 to improve the quality of PE in primary schools) [31] made it difficult to use funds flexibly to meet their specific needs. They also highlighted time-related constraints in delivering PE in a crowded curriculum and a lack of staff capacity to support off-site activities. Some school staff expressed concern about the sustainability of quality PA provision due to over-reliance on a small number of unspecialised staff.

In contrast, many schools identified key enablers that supported an active school environment. These included strong leadership support, peer collaboration among staff, and targeted efforts to increase PA participation among some groups. For example, girls-only sports clubs, free clubs for pupils eligible for the pupil premium, and inclusive competitions adapted for children with SEND. Expanding the range of sporting opportunities (e.g., access to swimming facilities and inclusive inter-school competitions) through external partnerships with local sports networks, charities, and community providers was welcomed by several schools. Some schools also described themed health weeks and event days to promote healthy lifestyles.

## Discussion

This study aimed to assess the extent to which Greater London state-funded primary schools implement the WHO’s whole-school approach to PA. WHO guidance identifies six key domains and recommends that effective school‑based PA interventions be multicomponent, addressing at least two of these domains for maximum impact [4]. Our quantitative survey found that two-thirds of schools responding to our survey reported implementing at least one practice within each of the six WHO domains, particularly through structured PA opportunities. This reflects broad provision that promotes physically active school environments. However, despite three-quarters of responding primary schools reporting that they had a PA policy in place, implementation in practice varied within each domain. This suggests that school PA policies may not fully implement WHO-aligned practices, which could contribute to persistently low PA levels among children.

Schools more commonly reported implementing items relating to QPE (Domain 1), extracurricular activities (Domain 3), and physically active breaks during the school day (Domain 4). This indicates that some WHO domains may be more feasible to implement in practice as they align closely with established school routines and timetabled activities. Curricular PE remained the cornerstone of school-based PA in nearly all schools. However, only around half of the schools reported meeting the curricular recommendation of an average of two hours of timetabled PE per week. Nationally, the Youth Sport Trust’s annual report indicated that primary schools provided an average of around 93 minutes per week in 2022 [32]. This shortfall may reflect barriers identified in previous research and our free-text responses, including an overcrowded and rigid curriculum, schools describing themselves as “*time poor,*” and teachers’ lack of confidence and expertise in delivering PE [33,34]. Many schools reported that specialist PE teachers or coaches either delivered PE or assisted class teachers, but access to specialist staff often depended on school budgets. Free‑text comments also showed variation in capacity across schools, with some relying on one or two staff members or coaches to drive provision, while others described strong leadership support and partnerships, pointing to the importance of whole‑school capacity for maintaining PA provision. These findings echo the Royal Society for Public Health’s recent recommendations, which call for embedding PA into routine teaching through structural support, such as staff training to support pupils’ mental and physical health, and flexible funding to address local barriers, without adding to teachers’ workload [35].

Beyond the curriculum, other school-based opportunities for PA were encouraging, but issues of equitable access to facilities were apparent. Scheduled breaks such as recess and lunch offered important PA opportunities, though access to outdoor spaces varied: playgrounds and portable equipment were widely available in most schools, but fewer than half had access to playing fields. This likely reflects the urban location of our sample, where space constraints and the cost of developing and maintaining large playing fields can limit availability [11]. Nearly all schools offered extracurricular PA, particularly after school. While after-school programmes can help reduce pupils’ sedentary time [36,37], extracurricular activity typically relies on teachers volunteering to provide these opportunities, parental support, and potential costs for pupils to participate, which may exclude some children from families with low incomes [38,39]. One school illustrated how such inequalities manifest in practice, explaining that “*the fitness of children in this school compared to previous schools in more disadvantaged areas is noticeably different. …this takes financial investment and was not as available in previous schools, where the children were also less exposed to this [better‑resourced environment] outside of school.*” Furthermore, schools are increasingly expected to ensure equitable access to PA opportunities [40], including before school, on weekends, and during holidays, but provision during these times was reportedly low in our study.

Active travel initiatives were widely implemented, with schools reporting infrastructure to support walking or cycling. The physical environment, neighbourhood walkability, and parental perceptions of traffic and pedestrian safety significantly influence uptake of active school travel [41,42], with children’s school journey risks and experiences differing across neighbourhood contexts and socio-economic groups [43,44]. The relatively low promotion of ‘Park and Stride’ schemes in our sample may reflect the reach of the ‘School Streets’ programme, which by late 2024 had been implemented in over 38.2% of primary schools, temporarily restricting traffic outside schools during drop-off and pick-up times [45]. As such, additional promotion of the Park and Stride scheme may have been considered unnecessary.

More than two-thirds of schools in our study reported incorporating PA into general classroom teaching. A meta-analysis of 16 studies showed that movement breaks during lessons improved on-task classroom behaviour, although evidence was mixed regarding the influence of this type of intervention on cognitive development, PA levels, and academic outcomes. This may be partly due to the heterogeneity of intervention content and the variety of outcome assessment tools, such as those used to measure PA levels [46]. More than half of schools reported participation in active mile initiatives, which have been associated with improved cardiorespiratory fitness and positive attitudes to PA among pupils [30]. However, there are concerns that such initiatives may replace rather than supplement curricular PE time, and adherence is variable and often unmonitored [47].

Notably, most schools reported implementing inclusive PA that supports children with SEND. In free-text responses, these schools highlighted their participation in inclusive sporting fixtures, provision of one-to-one staff support and training, and the use of adapted play equipment. One school acknowledged a barrier to equitable access, noting that “*access for SEND pupils in sport can be limited due to the training of coaches; parents would feel more willing to send children with needs to sessions if they were confident in their child’s needs being met.*” Further investigation beyond self-reported data would provide greater insights into the quality, accessibility, and consistency of such provision.

### Strengths and limitations

A key strength of our study is that it is the first, to our knowledge, to examine schools’ physical activity (PA) policies and practices assessed against global recommendations for promoting a whole-school approach to PA. Another major strength is the use of a piloted survey instrument informed by the WHO’s whole-school PA promotion framework, enabling structured data collection across all six recommended domains. Unlike national surveys, which are the main sources of data assessing children’s engagement with and attitudes to PA [6], our approach captured the presence and variation of institutional-level PA policies, infrastructure, and practices aligned with the WHO framework. The distribution of our survey to schools in an ethnically and socioeconomically diverse urban conurbation is another important strength of our study. Our findings may be representative of other major, diverse conurbations, as we received responses from at least one school in every Greater London borough with varying levels of deprivation.

Our study’s strengths should be considered alongside its limitations. Our response rate may be considered low; however, challenges with obtaining survey responses from schools are widely recognised [48]. Input from both classroom and leadership staff captured diverse perspectives, but role-specific knowledge gaps (e.g., policy-level decisions) may have introduced inconsistencies or misreporting, potentially affecting the reliability of responses across schools. A significant difference between responding and non-responding schools in the proportion of children eligible for free school meals, which serves as a proxy for socioeconomic status, may limit the generalisability of our results. Schools with fewer disadvantaged pupils typically have greater resources and capacity to implement PA-supportive policies, which could inflate our estimates of implementation relative to the wider population. Additionally, the number of questions included under each domain varied, with three domains assessed using only a single question, while others included two or three questions. Domains with fewer questions may have limited the depth and rigour of evaluation.

Future surveys should target respondents with role-specific expertise or adopt consensus-based responses using a more extensive survey. They could also refine and expand domain-specific indicators by incorporating rating scales or quality assessments. A richer dataset could be obtained by integrating qualitative methods and observational approaches alongside survey responses, thereby providing clearer and more actionable criteria for policymakers and school authorities to monitor progress and support efforts to overcome persistent barriers, such as limited curricular PE time and inadequate access to facilities. Our findings further underscore the need to identify these barriers among more deprived schools, which were less likely to respond and may face distinct challenges in implementing whole-school PA approaches. Nonetheless, the study offers valuable insights for public health practitioners, education policymakers, and in-school providers seeking to embed equitable and sustainable approaches to promoting PA in primary schools.

## Conclusion

To our knowledge, this is the first study to assess whether the WHO guidelines for a whole-school approach to PA promotion are implemented in a large, urban, ethnically diverse conurbation. The widespread engagement in structured PA domains demonstrates that schools recognise the value of PA. Our study provides an operationalised assessment of the active school environment, which forms the basis for future research to explore its role in supporting other outcomes such as mental health and academic performance. By identifying both the breadth and variability of domain implementation, our study contributes to evaluating whole-school approaches and active school environments in practice, aligning with WHO guidance.

## Supporting information

S1 Table WHO domains and definitions. (PDF)

S1 File STROBE Checklist in reports of cross-sectional studies. (PDF)

S2 File School Survey. (PDF)

S2 Table Questions extracted from survey. (PDF)
