# Supplementary material for: Real-world evidence of physical activity practices and policies in Greater London primary schools: A cross-sectional survey
Source: PLoS One. 2026 Jul 10;21(7):e0352283. doi: 10.1371/journal.pone.0352283 (PMC13354065; doi:10.1371/journal.pone.0352283)
Supplement: S1 Table — (PDF) [file pone.0352283.s001.pdf]

S1 Table. WHO domains and definitions.[1]

WHO Domain		Definition
1	QPE (Quality Physical Education)	Where physical education (PE) is planned, progressive, and ensures inclusive learning experience that forms part of the curriculum throughout all school years.
2	Active travel	Walking, cycling, or other means of active travelling to school as an alternative to motorised transport (i.e., cars) but excluding public transport which usually requires walking or cycling to the mode of transport.
3	Physical activity (PA) opportunities before and after school	Organised physical activity opportunities taking place outside of the curriculum which can be delivered in school by staff, peer leaders, or volunteers, or in the local community through externally funded organisations.
4	Opportunities for PA at recess and lunch	Physical activity opportunities for pupils in all year groups during playtimes/breaks.
5	Active classrooms	To incorporate physical activity (e.g., stretching, jumping) any time during the school day in one or more lessons.
6	PA for those with additional needs	Physical activity provision should accommodate all pupils regardless of stage in development, fitness, skill level, health, body size, or maturity.

[1]WHO. Promoting physical activity through Schools: A Toolkit. Geneva: World Health Organisation; 2021.
