# Supplementary material for: Real-world evidence of physical activity practices and policies in Greater London primary schools: A cross-sectional survey
Source: PLoS One. 2026 Jul 10;21(7):e0352283. doi: 10.1371/journal.pone.0352283 (PMC13354065; doi:10.1371/journal.pone.0352283)
Supplement: S2 File — (PDF) [file pone.0352283.s003.pdf]

S2 File. School Survey.

Imperial College London

HAPPY

Health & Activity of Pupils in the Primary Years

School Activity Policies and Practices Survey

We are a research team at Imperial College London who are investigating how school environments may impact children's health and wellbeing.

We would like to find out about your school's activity policies and practices through this short survey - it should only take around 10 minutes to complete!

All primary schools in Greater London are being sent this survey. Your responses will help us to better understand how school environments may be able to encourage children to be active throughout the school day.

Your responses will be treated in strict confidence and any data that we use from the survey will not identify you or your school.

Thank you!

This questionnaire specifies all year groups (Reception to Year 6). Please only answer according to the year groups relevant to your school

Section A: About your school

1. Name of your school

____

2. What is your school's postcode? ______________________________

3. Your name in full

____

4. Your email address

____

5. What is your role at the school?
   - ○ Headteacher
   - ○ Teacher
   - ○ Administrator
   - ○ Other (please specify below what your role is at the school):

____

6. What are the school day **start** and **finish** times for the year groups at your school?

	Start time	Finish time
Reception		
Year 1		
Year 2		
Year 3		
Year 4		
Year 5		
Year 6		

S2 File. Continued

7. What are the break and lunch times for the year groups at your school?

	Morning (e.g., 10-10:30am)	Lunch (e.g., 12-1pm)	Afternoon (e.g., 2-2:30pm)	Any other break times? *Please leave blank if no other break times*
Reception				
Year 1				
Year 2				
Year 3				
Year 4				
Year 5				
Year 6				

Section B: Questions about physical activity at your school

8. Does your school have a physical activity policy?
   - ○ Yes
   - ○ No

9. Does your school have any of the following in place for facilitating physical activity? *Please select all that apply*

Have a designated physical activity coordinator	☐
Training of staff with regard to physical activity	☐
Active travel plan (where children are encouraged to walk (e.g., walking school bus), cycle or use public transport to get to school)	☐
A 'Park and Stride' (parents park their cars or stop and drop their children off away from the school and children walk the remainder of the journey)	☐
Active mile campaign/initiatives (e.g., The Daily Mile, Marathon Kids etc.)	☐
An entrance to the school for pedestrians/cyclists separate from cars	☐
Playtime activity	☐
Curricular physical education	☐
Out of school hours physical activities	☐
Physical activities for staff	☐
Raising the profile of physical activity	☐
School sports partnerships and community links	☐
None of these	☐
Other (please specify below):	

---

10. How many hours of physical education do the children usually have **per week**?

	Less than 1 hour	1 hour to <2 hours	2 hours to <3 hours	3 hours to <4 hours	4 hours or more
Reception	○	○	○	○	○
Year 1	○	○	○	○	○
Year 2	○	○	○	○	○
Year 3	○	○	○	○	○
Year 4	○	○	○	○	○
Year 5	○	○	○	○	○
Year 6	○	○	○	○	○

11. Who **usually** teaches physical activity at the school?
   - ○ Specialist PE teacher from inside the school
   - ○ Teacher (not PE specialist)
   - ○ Adult specialist from outside of the school

   Other (please specify): ______________________________

S2 File. Continued

12. Does your school have access (either on-site or off-site) to the following and are they fit for purpose?

	Access to facility / equipment?		Is the facility/equipment fit for purpose?		
	Yes	No	Yes	No	N/A
A specific hall for gym or sports (indoors)	○	○	○	○	○
A shared facility used for sports activities (indoors)	○	○	○	○	○
A sports or football field/pitch (on school grounds)	○	○	○	○	○
A school playground	○	○	○	○	○
A swimming pool	○	○	○	○	○
A local park or playground (off school ground) available to use	○	○	○	○	○
Changing facilities (purpose built)	○	○	○	○	○
Sports equipment (e.g., gymnastics equipment)	○	○	○	○	○

13. Are the school grounds generally suitable for…

	Yes	No
Sport (organised and not)	○	○
Informal games (e.g., kickabout, frisbee, etc.)	○	○
General play	○	○

14. In your opinion, to what extent do you agree or disagree that promotion of physical activity is supported by…

	Strongly agree	Agree	Neither agree nor disagree	Disagree	Strongly disagree
Senior Leadership Team (including school governors, headteacher, deputy headteacher)	○	○	○	○	○
Teachers and/or teaching support staff	○	○	○	○	○
Pupils	○	○	○	○	○

15. When does your school (or any other organisation) provide any extracurricular physical activity or sports programmes for children at your school?

	Yes	No
Before school	○	○
During lunch breaks	○	○
After school	○	○
During weekends	○	○
Any other time (please specify): --		

S2 File. Continued

16. Do children have access to the following facilities/equipment for physical activities during breaks and lunch times?

	Yes	No
Playground	○	○
Playing field	○	○
Hard court area (i.e., for tennis or basketball)	○	○
Permanent playground equipment	○	○
Portable playground equipment (e.g., hoops, tennis rackets and balls, cricket bats and balls, football etc.)?	○	○
Other, please specify: ________________		

17. Are children given the opportunity to be physically active during lessons (e.g., where physical activity is incorporated into the lesson)? This excludes physical education (PE) lessons.
   - ○ Yes
   - ○ No

If yes, please provide one or two examples of how children are physically active during lessons (this does not include PE lessons).

____

____

____

18. Is there sufficient provision (staff/equipment) to support participation of children with specific disabilities or impairments in physical activities?
   - ○ Yes
   - ○ No

19. If any provision (staff/equipment) is provided to support participation for children with specific disabilities or impairments in physical activity, regardless of whether provision is sufficient, please specify below:

____

____

Section C: Final comments

20. Is there anything you would like to tell us about physical activity of children or promotion at your school? Is there anything that this questionnaire did not capture about physical activity at your school?

____

____

____

____

____
