# Supplementary material for: Real-world evidence of physical activity practices and policies in Greater London primary schools: A cross-sectional survey
Source: PLoS One. 2026 Jul 10;21(7):e0352283. doi: 10.1371/journal.pone.0352283 (PMC13354065; doi:10.1371/journal.pone.0352283)
Supplement: S2 Table — (PDF) [file pone.0352283.s004.pdf]

S2 Table. Questions extracted from survey relevant to each WHO domain.

WHO Domain	Survey question	Response to meet criteria
1. Quality physical education	• Q9. Does your school have any of the following in place for facilitating physical activity?<br>• Q10. How many hours of physical education do the children usually have per week?<br>• Q11. Who usually teaches physical activity at the school?	• Curricular physical education (PE)<br>• 2 hours or more<br>• Specialist PE teacher (inside or outside of school)
2. Active travel	• Q9. Does your school have any of the following in place for facilitating physical activity?	• Active travel plan<br>• Park and stride<br>• Separate pedestrian/cyclist entrance
3. Before- and after-school opportunities	• Q15. Does your school (or any other organisation) provide any extracurricular physical activity or sports programmes for children at your school during the following times?	• Before school<br>• After school<br>• During weekends
4. Physical activity opportunities during recess (breaks) and recreation time (lunch)	• Q9. Does your school have any of the following in place for facilitating physical activity?<br>• Q15. Does your school (or any other organisation) provide any extracurricular physical activity or sports programmes for children at your school during the following times?<br>• Q16. Do children have access to the following facilities/equipment for physical activities during breaks and lunch times?	• Playtime activity<br>• Lunchtime<br>• Playground<br>• Playing field<br>• Hardcourt area<br>• Permanent playground equipment<br>• Portable playground equipment
5. Active classrooms	• Q17. Are children given the opportunity to be active during lessons?<br>• Q9. Does your school have any of the following in place for facilitating physical activity?	• Yes<br>• Active mile
6. Inclusive for children with additional needs	• Q18. Is there sufficient provision for children with disabilities in physical activities?	• Yes
